# Editorial: Clinical therapy of brain tumors

**DOI:** 10.3389/fneur.2025.1610463

**Published:** 2025-05-23

**Authors:** Boyuan Huang, Huijun Zhao, Jun Yang, Fusheng Liu, Gerardo Caruso, Hongbo Zhang, Lin Chen

**Affiliations:** ^1^Second Department of Neurosurgery, Capital Medical University Electric Power Teaching Hospital/State GridBeijing Electric Power Hospital, Beijing, China; ^2^Beijing Tiantan Hospital, Capital Medical University, Beijing, China; ^3^University Hospital of Policlinico G. Martino, Messina, Italy; ^4^Second Affiliated Hospital of Nanchang University, Nanchang, China

**Keywords:** brain tumor treatment, radiotherapy, chemotherapy, immunotherapy, neurosurgery

Brain tumors are devastating diseases, accounting for a significant proportion of cancer-related mortality and morbidity in both adults and children. Over the past few years, treatment for brain tumors have witnessed dramatic progress. This is largely due to advances in surgical skills, more rational and personalized radiotherapy and chemotherapy, and continually updated immunotherapies that modulate the immune microenvironment or specifically target tumor cells. This editorial summarizes significant findings on surgery, radiotherapy, chemotherapy, immunotherapy and other treatment published in the Research Topic, Clinical Therapy of Brain Tumors, Frontiers in Neurology, emphasizing their impact on advancing the understanding and treatment of brain tumors. The current Research Topic includes 17 papers, among which 7 were considered as Original Research, with 3 Reviews and 7 case reports.

## 1 Surgical treatment for brain tumors

Prior to surgery, various imaging techniques can provide information about the nature of the tumor, including its location, blood supply, metabolic status, and key surrounding functional areas. Furthermore, magnetoencephalography (MEG) and guided transcranial magnetic stimulation (nTMS) have emerged as new tools for the localization of important areas of function. It was found that the application of nTMS could improve surgical total resection rates for low-grade gliomas (LGGs) by approximately 16% and increase median progression-free survival (PFS) from 15.4 months to 22.4 months (1, 2). Moreover, application of intraoperative assistive technology such as intraoperative navigation, intraoperative magnetic resonance imaging, intraoperative ultrasound and intraoperative fluorescence, which can assist the operator in effectively localizing lesion and extent of the tumor, can also increase the success of the operation. In addition, intraoperative electrophysiological monitoring techniques and intraoperative awakening technique can help the surgeon localize the important functional areas of the brain, which play a significant role in protecting the function of the important functional areas of the patient. Duan et al. highlighted that with proper management of the sagittal sinus and protection of the associated veins, the surgical treatment strategy for the “radical” resection of parasagittal sinus meningioma is effective, safe and simple to perform. He et al. reviewed the clinical characteristics and treatment outcomes of 14 patients with polymorphic low-grade neuroepithelial tumor of the young and highlighted that stereoelectroencephalography was pivotal for cases with unclear lateralization, aiding in identifying the link between the tumor and seizures. They suggested that following established epilepsy surgery protocols for brain tumor management, early intervention and extended resection can improve the rate of postoperative seizure freedom. Gui et al. reported a rare case of neurocytoma originating from cranial nerve V.

## 2 Radiotherapy for brain tumors

Radiotherapy is primarily used to treat malignant neoplasms, including gliomas and brain metastases, benign neoplasms that are not amenable to complete resection, such as meningiomas, as well as neoplasms that appear sensitive to radiotherapy, such as germ cell tumors. In recent years, with the development in science and technology, a multitude of novel techniques have been employed in radiotherapy, such as proton radiotherapy and heavy ion radiotherapy (HIRT), which has led to significant improvement in patient prognoses. Compared with conventional radiotherapy, proton radiotherapy, with more precise dose distribution, is able to further protect the surrounding normal tissues without loss of clinical efficacy (3). Although the current evidence for proton radiotherapy in brain tumors is limited, it still shows good prospects for application (4). In the current Research Topic, Palenzuela et al. studied acute toxicity of chemotherapy in central nervous system germ cell tumor patients according to age. Li et al. found that adjuvant beam radiation therapy could enhance overall survival (OS) in younger primary single intracranial atypical meningioma patients.

## 3 Systemic therapy for brain tumors

Systemic anti-tumor agents broadly include traditional cytotoxic chemotherapeutic agents, molecularly targeted agents and immunotherapeutic agents, mainly for high grade gliomas, brain metastases, lymphomas, etc. The molecularly targeted agents includes IDH1/2 mutant inhibitors, BRAF/MEK inhibitors, NTRK fusion inhibitors, MET kinase inhibitors, antiangiogenic drugs, etc. Vorasidenib, an oral IDH1/2 mutant inhibitor, was shown in a phase III study to significantly increase progression-free survival (PFS) in patients with grade 2 IDH-mutated oligodendroglioma or astrocytoma (5). In the Research Topic, Bao et al. made a retrospective study of chemotherapy strategies for adults with IDH-wildtype glioblastoma (GBM). In addition, National Comprehensive Cancer Network Guidelines (NCCN) recommended that patients with NTRK-compatible gliomas should be treated with NTRK fusion inhibitors, such as larotrectinib and entrectinib (6, 7). Although it can increase PFS of patients, bevacizumab, the first antiangiogenic drug approved by the FDA and recommended by NCCN to treat recurrent GBM, still failed in prolonging OS (8). Currently, the most promising immunotherapeutic agents against brain tumors are only immune checkpoint inhibitors, such as PD-1/PD-L1 antibodies (Pembrolizumab, Nivolumab). Although failed in phase III clinical trial against recurrent GBM (9), anti-PD-1 immunotherapy still showed efficiency in increasing GBM patient OS as neoadjuvant systemic therapy (10).

Other forms of immunotherapy, including tumor vaccine therapy, chimeric antigen receptor T (CAR-T) cell therapy and oncolytic virus therapy, have also shown promise in the treatment of brain tumors. A dendritic cell vaccine called DCVax-L showed obvious efficacy in clinical trials against primary GBM, with a median OS of 23.1 months (11). G47Δ, based on human herpes simplex virus, had markedly improved 1-year survival rate to 84.2%, and median OS to 20.2 months for residual or recurrent GBM patients (12). In 2021, G47Δwas approved for marketing in Japan for the treatment of residual or recurrent GBM. According to these above results, the Society for Immunotherapy Of Cancer (SITC) published a consensus statement that immunotherapy can be used as a salvage treatment option for gliomas patients after conventional treatment, which required further optimization of combination therapy (13). In the current Research Topics, Chen et al. performed a literature review about the immune microenvironment and immunotherapy for chordoma. In addition, Pu et al. unveiled substantial involvement of MAP2K3 in gliomas, indicating the potential of the enzyme to serve as a prognostic biomarker related to immunity. Through the regulation of the infiltration of immune cells, MAP2K3 can affect the prognosis of patients with glioma. Shen et al. performed a multivariate Cox proportional hazards regression analyses to identify independent prognostic variables for GBM patients with synchronous metastasis (SM). They found that radiotherapy, chemotherapy, and surgery constitute an effective treatment regimen for patients with SM. Luo et al. performed a literature review in which they summarized the latest advancements in understanding the molecular mechanisms that regulate regulated cell death in glioma and explore the interconnections between different cell death processes. Palenzuela et al. compared the tolerance of chemotherapy across age-groups within the SIOP-CNS-GCT-II trial.

## 4 Tumor treating fields for brain tumors

Tumor treating fields (TTF) is a new type of therapy applying physics that works by delivering low-intensity, medium-frequency alternating electric fields through a patch applied to the scalp. The fundamental mechanism by which TTF exerts its therapeutic effect involves anti-tumor cell mitosis, suppression of DNA damage repair, disruption of tumor cell migration, and potentiation of anti-tumor immune responses (14). The versatility of this therapeutic modality renders it a promising candidate for concurrent utilization with chemotherapy, radiotherapy, anti-angiogenic therapy, and immunotherapy (15). The results from a prospective, single-arm, phase I clinical trial demonstrated that the combination of TTFields and chemotherapy may offer survival benefits for recurrent GBM patients (16). Moreover, TTF in combination with TMZ and pembrolizumab adjuvant therapy could improved the median OS of primary GBM patients to 24.8 months, with a two-year OS rate of 52.4% (17), which supported the safety and efficiency of TTF in combination with chemotherapy and immunotherapy, and thus requires further exploration. Currently, TTF has been recommended by the NCCN for combined administration with TMZ for primary GBM patients after surgery or radiotherapy, or alone for recurrent GBM patients (15).

## 5 Conclusion

Recent advancements in surgical assistive technologies have led to significant progress in the field of neurosurgery. These technologies play a crucial role in protecting vital neurological functions, enabling neurosurgeons to more accurately determine the margin between tumor and normal brain tissue. Additionally, these technologies have also contributed to increasing the extent of brain tumor resection and, consequently, enhancing the prognosis for brain tumor patients. Radiotherapy constitutes a critical localized treatment modality for gliomas, playing a pivotal role in the management of inoperable cases and/or for local disease control. Continuous advancements in novel radiotherapy techniques have led to enhanced control of brain tumors while concurrently reducing adverse effects. Drug therapy, immunotherapy and TTF, emerged as critical tools in the control of brain tumors, have also demonstrated efficacy in the management of brain tumors. Consequently, the future holds more promising significant advancements in the treatment of brain tumors, which will further improve patient outcomes.
